# Supplementary material for: YAP localization mediates mechanical adaptation of human cancer cells during extravasation in vivo
Source: bioRxiv. 2023 Nov 16:2023.11.14.567015. Preprint. [Version 1] doi: 10.1101/2023.11.14.567015 (PMC10705547; doi:10.1101/2023.11.14.567015)
Supplement: Supplement 1 [file NIHPP2023.11.14.567015V1-supplement-1.pdf]

bioRxiv preprint doi: https://doi.org/10.1101/2023.11.14.567015; this version posted November 16, 2023. The copyright holder for this preprint (which was not certified by peer review) is the author/funder. This article is a US Government work. It is not subject to copyright under 17 USC 105 and is also made available for use under a CC0 license.

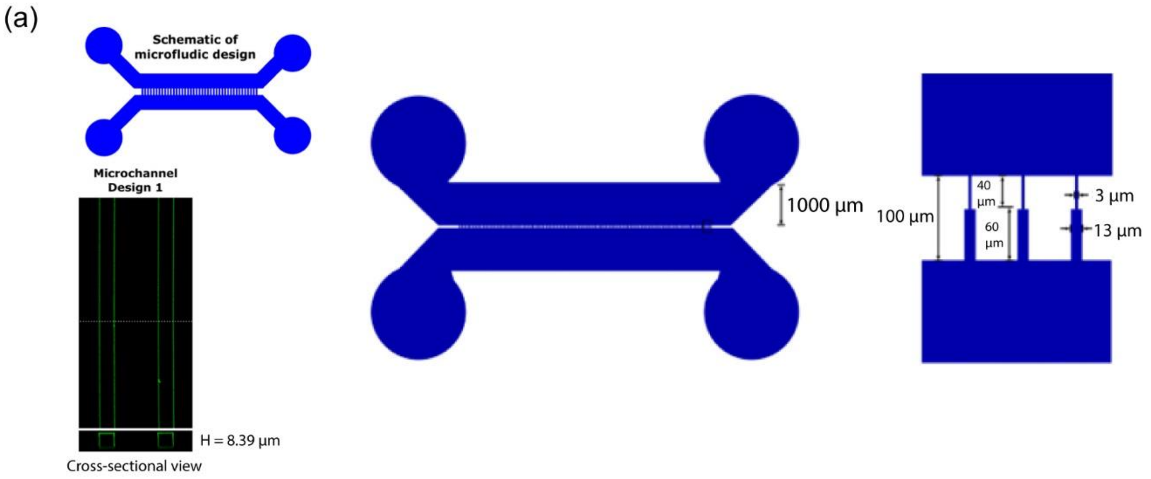

Supplementary Figure 1:

(a) Scheme of extravasation mimicking device design

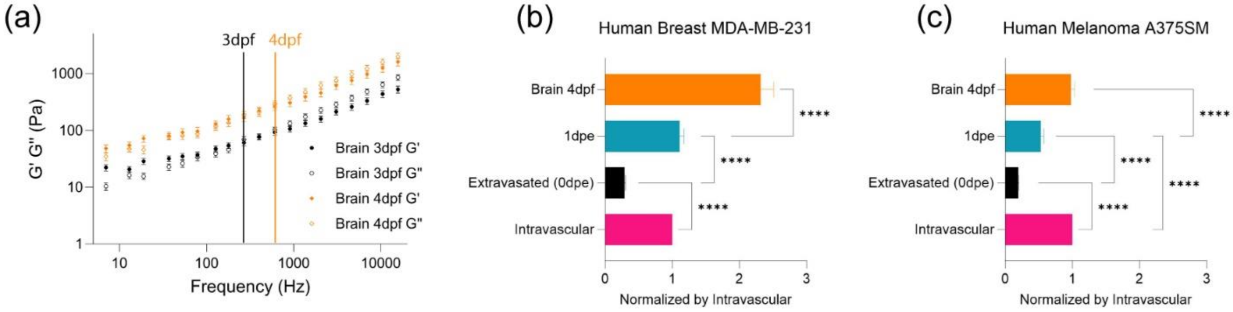

Supplementary Figure 2:

bioRxiv preprint doi: https://doi.org/10.1101/2023.11.14.567015; this version posted November 16, 2023. The copyright holder for this preprint (which was not certified by peer review) is the author/funder. This article is a US Government work. It is not subject to copyright under 17 USC 105 and is also made available for use under a CC0 license.

(a) log-log plot of *in vivo* brain tissue mechanics (elastic modulus, G', and viscous modulus, G'') and frequencies (7Hz to 15kHz) in the function of '3dpf' (number of fish = 5, n = 132) and '4dpf' (number of fish = 5, n = 154). Error bars in standard of error. Crossover frequency is assigned for each condition to point out the frequency where G'' becomes more dominant than G'. (b) Normalized bar graph of complex modulus for 'Breast (Intravascular)', 'Breast (0dpe)', 'Breast (1dpe)', and 'Brain 4dpf' in respect to 'Breast (Intravascular)' based on 19 different frequencies from 7Hz to 15kHz. **** p<0.0001, paired two-tailed t-tests. (c) Normalized bar graph of complex modulus for 'Melanoma (Intravascular)', 'Melanoma (0dpe)', 'Melanoma (1dpe)', and 'Brain 4dpf' in respect to 'Melanoma (Intravascular)' based on 19 different frequencies from 7Hz to 15kHz. **** p<0.0001, paired two-tailed t-tests.

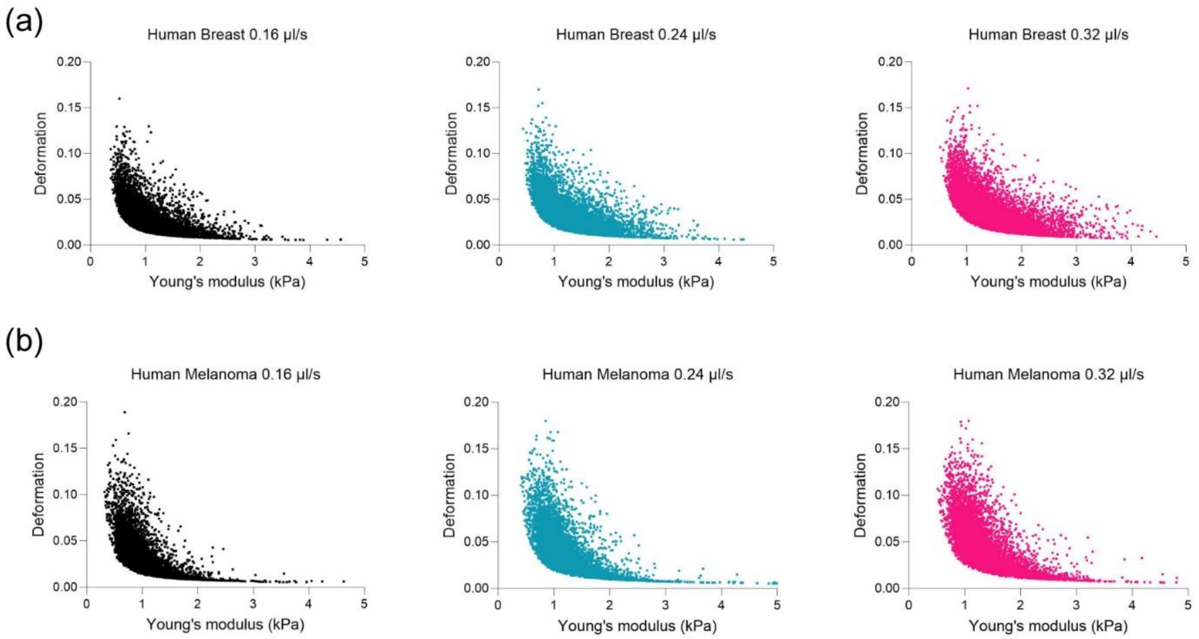

Supplementary Figure 3:

(a) Deformation and Young's modulus (kPa) plot of human breast cancer cell at different flow velocities, 0.16 µl/s (n=4906), 0.24 µl/s (n=7398), and 0.32 µl/s (n=9506) (b) Deformation and

bioRxiv preprint doi: https://doi.org/10.1101/2023.11.14.567015; this version posted November 16, 2023. The copyright holder for this preprint (which was not certified by peer review) is the author/funder. This article is a US Government work. It is not subject to copyright under 17 USC 105 and is also made available for use under a CC0 license.

Young's modulus (kPa) of human melanoma at different flow velocities, 0.16 µl/s (n= 3954), 0.24 µl/s (n= 6284), and 0.32 µl/s (n= 8442).

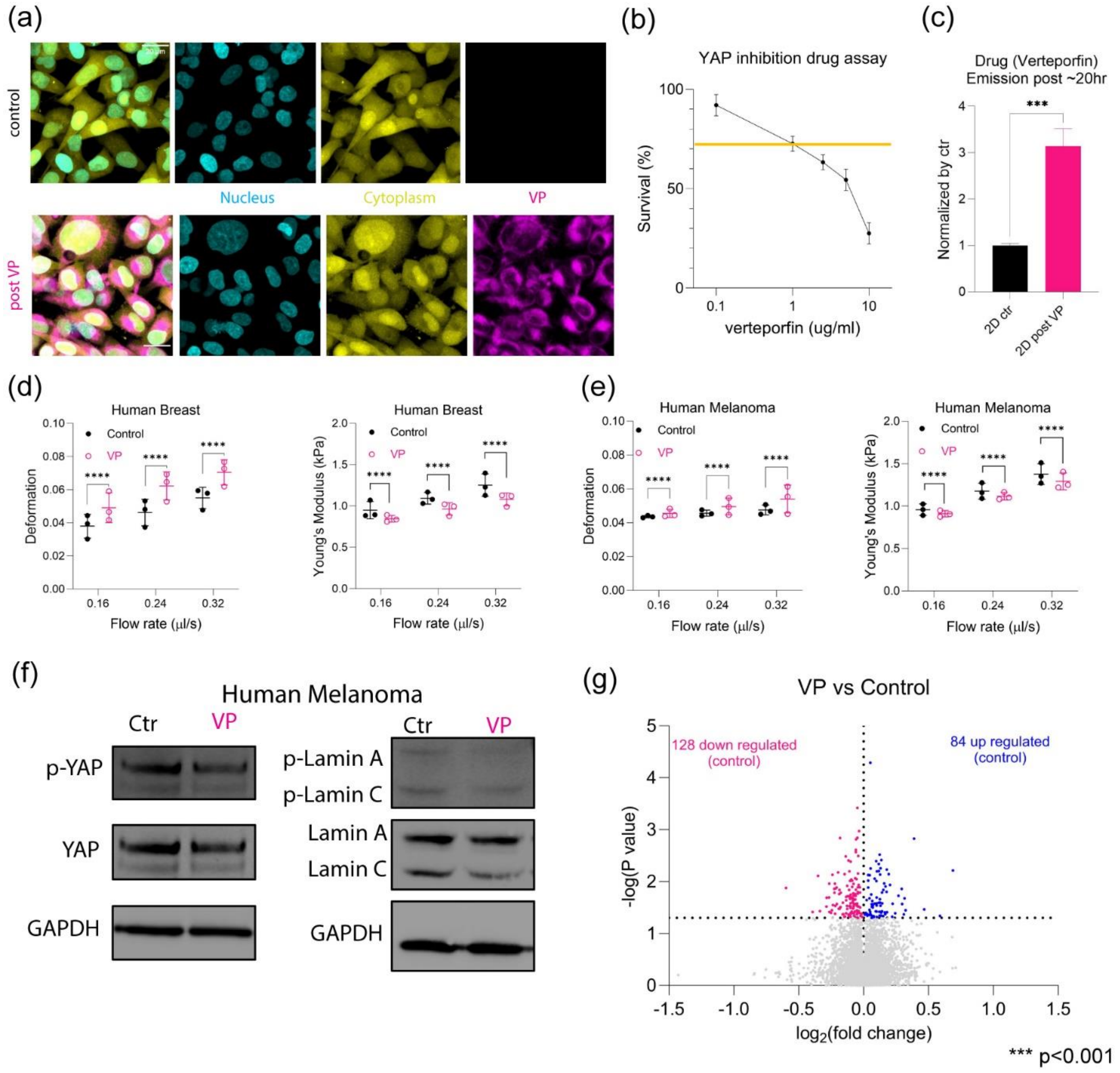

Supplementary Figure 4:

(a) Live imaging of cells between control/DMSO and verteporfin (VP) treated human breast cancer cells (b) YAP inhibition (VP) drug assay from 0.01 to 10 µg/ml (N=3) (c) Emission intensity of VP between control/DMSO cells and post VP treated cells (after ~20hr) (N=3). ***

bioRxiv preprint doi: https://doi.org/10.1101/2023.11.14.567015; this version posted November 16, 2023. The copyright holder for this preprint (which was not certified by peer review) is the author/funder. This article is a US Government work. It is not subject to copyright under 17 USC 105 and is also made available for use under a CC0 license.

p<0.001, paired two-tailed t-tests. (d) Deformation comparison for human breast cancer cells between control/DMSO (N=3; per each replicate more than 1000 cells) and VP (N=3; per each replicate more than 1000 cells) at 0.16 µl/s, 0.24 µl/s, and 0.32 µl/s. **** p<0.0001, paired two-tailed t-tests. Young's modulus (kPa) comparison for human breast cancer cells between control/DMSO (N=3; per each replicate more than 1000 cells) and VP (N=3; per each replicate more than 1000 cells) at 0.16 µl/s, 0.24 µl/s, and 0.32 µl/s. **** p<0.0001, paired two-tailed t-tests. (e) Deformation comparison for human melanoma between control/DMSO (N=3; per each replicate more than 1000 cells) and VP (N=3; per each replicate more than 1000 cells) at 0.16 µl/s, 0.24 µl/s, and 0.32 µl/s. **** p<0.0001, paired two-tailed t-tests. Young's modulus (kPa) comparison for human melanoma between control/DMSO (N=3; per each replicate more than 1000 cells) and VP (N=3; per each replicate more than 1000 cells) at 0.16 µl/s, 0.24 µl/s, and 0.32 µl/s. **** p<0.0001, paired two-tailed t-tests. (f) Representative western blot of total YAP with phosphorylated YAP and total Lamin A/C with phosphorylated Lamin A/C for human melanoma (N=3). (g) Volcano plots, showing up-regulated and down-regulated proteins for control/DMSO.

bioRxiv preprint doi: https://doi.org/10.1101/2023.11.14.567015; this version posted November 16, 2023. The copyright holder for this preprint (which was not certified by peer review) is the author/funder. This article is a US Government work. It is not subject to copyright under 17 USC 105 and is also made available for use under a CC0 license.

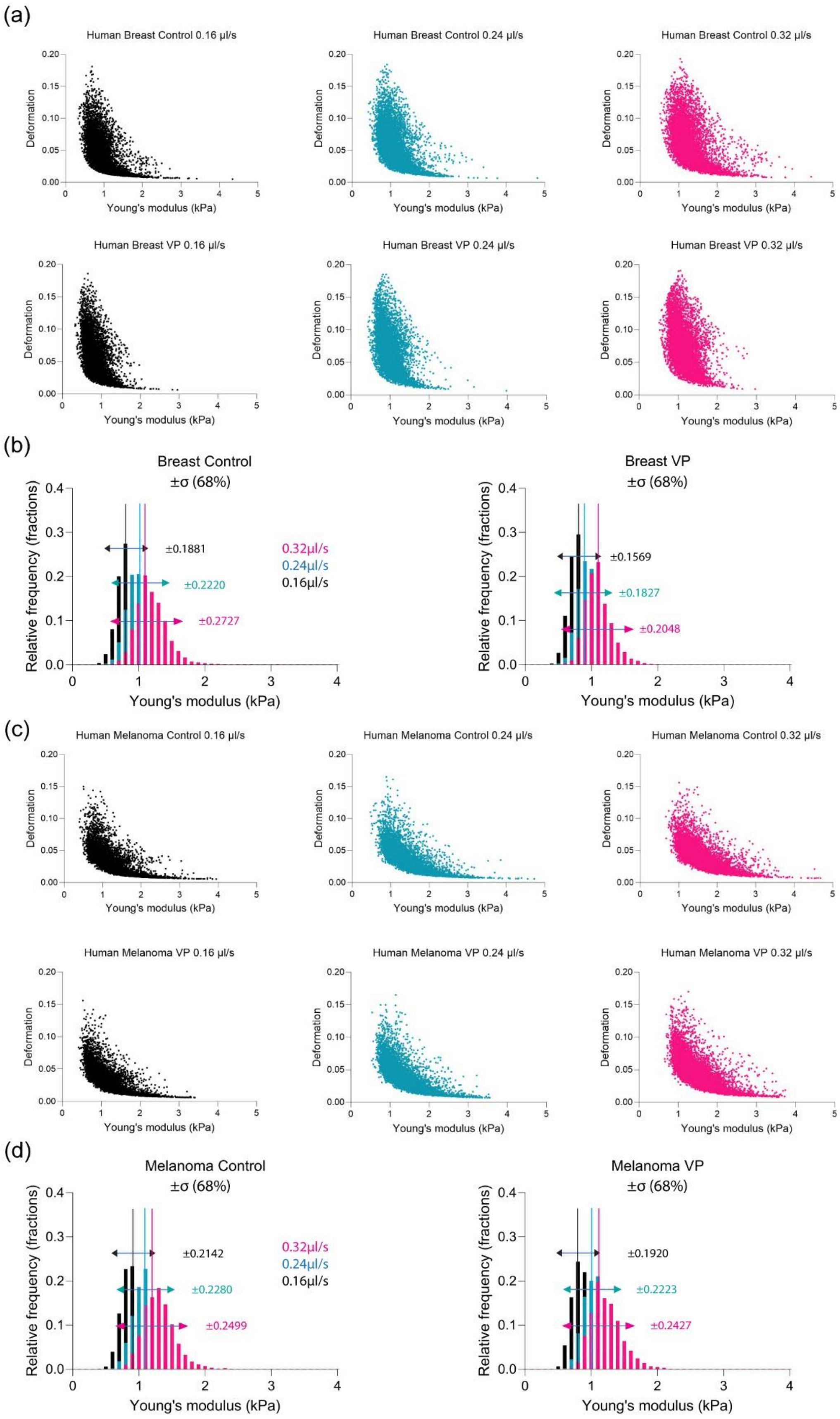

bioRxiv preprint doi: https://doi.org/10.1101/2023.11.14.567015; this version posted November 16, 2023. The copyright holder for this preprint (which was not certified by peer review) is the author/funder. This article is a US Government work. It is not subject to copyright under 17 USC 105 and is also made available for use under a CC0 license.

Supplementary Figure 5:

(a) Deformation and young's modulus (kPa) plot of human breast cancer cell control/DMSO at different flow velocities, 0.16 µl/s (n=6495), 0.24 µl/s (n=8401), and 0.32 µl/s (n=8712) with a fit based on 'deformation=(slope)/young's modulus. Deformation and young's modulus (kPa) plot of human breast cancer cell VP at different flow velocities, 0.16 µl/s (n=8734), 0.24 µl/s (n=10095), and 0.32 µl/s (n=10637) with a fit based on 'deformation=(slope)/young's modulus. (b) Normalized histogram of human breast cancer cell for young's modulus (kPa) between control/DMSO and VP at different flow velocities, 0.16 µl/s (control n=6495, VP n=8734), 0.24 µl/s (control n=8401, VP n=10095), and 0.32 µl/s (control n=8712, VP n=10637), with standard deviation (σ, ±68%). (c) Deformation and young's modulus (kPa) plot of human melanoma control at different flow velocities, 0.16 µl/s (n=9979), 0.24 µl/s (n=9991), and 0.32 µl/s (n=9928) with a fit based on 'deformation=(slope)/young's modulus. Deformation and young's modulus (kPa) plot of human melanoma VP at different flow velocities, 0.16 µl/s (n=9992), 0.24 µl/s (n=9991), and 0.32 µl/s (n=9988) with a fit based on 'deformation=(slope)/young's modulus. (d) Normalized histogram of human melanoma for young's modulus (kPa) between control/DMSO and VP at different flow velocities, 0.16 µl/s (control n=9979, VP n=9992), 0.24 µl/s (control n=9991, VP n=9991), and 0.32 µl/s (control n=9928, VP n=9988), with standard deviation (σ, ±68%).

Supplementary Table 1: Calculated stress for channel width of 30 µm and Cellcarrier solution

Cellcarrier – 30 µm	Pa
0.32 µl/s	1890
0.24 µl/s	1420
0.16 µl/s	947

Stress under laminar flow based on Poiseuille's law

bioRxiv preprint doi: https://doi.org/10.1101/2023.11.14.567015; this version posted November 16, 2023. The copyright holder for this preprint (which was not certified by peer review) is the author/funder. This article is a US Government work. It is not subject to copyright under 17 USC 105 and is also made available for use under a CC0 license.

$$Pressure = \frac{8Q\eta l}{\pi r^4}$$

Where Q is flow velocity, η is viscosity of fluid, l is the channel length, r is radius of channel

CellCarrier = $3.92*10^{-3}$ (Pa*s)

Supplementary Movie 1:

Extravasation moment of human breast cancer cells (MDA-MB-231) at zebrafish brain after injection of cells into the circulation of zebrafish, Tg(flk:mCherry/MRC1a:EGFP), at 2dpf. Blood vessel (flk) is in magenta, vein (MRC1a) is in yellow, and human breast cancer cells are in blue.

Supplementary Movie 2:

Human breast cancer cells (MDA-MB-231) with bead migrating in narrow channel inside of microfluidic device.

Supplementary Movie 3:

Tracking extravasated human breast cancer cells (MDA-MB-231) at zebrafish brain right after extravasation from 3dpf/1dpi overnight to 4dpf/2dpi. Blood vessel (flk) and vein (MRC1a) are in grey while human breast cancer cells are in blue.

Supplementary Movie 4:

Live cell imaging of control/DMSO cancer cells with nucleus (H2B) in magenta and membrane dye (CellTracker) in green inside of extravasation mimicking microfluidics devices at different time interval.

bioRxiv preprint doi: https://doi.org/10.1101/2023.11.14.567015; this version posted November 16, 2023. The copyright holder for this preprint (which was not certified by peer review) is the author/funder. This article is a US Government work. It is not subject to copyright under 17 USC 105 and is also made available for use under a CC0 license.

Supplementary Movie 5:

Live cell imaging of VP (Yap inhibition) treated cancer cells with nucleus (H2B) in magenta and membrane dye (CellTracker) in green inside of extravasation mimicking microfluidics devices at different time interval.

Supplementary Movie 6:

Overnight movie after co-injecting control/DMSO (cyan) and VP (magenta) treated human breast cancer cells into circulation at 2dpf using Tg(flk:mCherry/MRC1a:EGFP); flk and MRC1a are labelled as grey.
